# A Stochastic Multi-Scale Model of HIV-1 Transmission for Decision-Making: Application to a MSM Population

**DOI:** 10.1371/journal.pone.0070578

**Published:** 2013-11-26

**Authors:** Lilit Yeghiazarian, William G. Cumberland, Otto O. Yang

**Affiliations:** 1 Department of Biomedical, Chemical & Environmental Engineering, University of Cincinnati, Cincinnati, Ohio, United States of America; 2 Department of Biostatistics, School of Public Health, University of California Los Angeles, Los Angeles, California, United States of America; 3 Departments of Medicine and Microbiology, Immunology, and Molecular Genetics, Geffen School of Medicine, University of California Los Angeles, Los Angeles, California, United States of America; National Institute for Public Health and the Environment, Netherlands

## Abstract

**Background:**

In the absence of an effective vaccine against HIV-1, the scientific community is presented with the challenge of developing alternative methods to curb its spread. Due to the complexity of the disease, however, our ability to predict the impact of various prevention and treatment strategies is limited. While ART has been widely accepted as the gold standard of modern care, its timing is debated.

**Objectives:**

To evaluate the impact of medical interventions at the level of individuals on the spread of infection across the whole population. Specifically, we investigate the impact of ART initiation timing on HIV-1 spread in an MSM (Men who have Sex with Men) population.

**Design and Methods:**

A stochastic multi-scale model of HIV-1 transmission that integrates within a single framework the in-host cellular dynamics and their outcomes, patient health states, and sexual contact networks. The model captures disease state and progression within individuals, and allows for simulation of therapeutic strategies.

**Results:**

Early ART initiation may substantially affect disease spread through a population.

**Conclusions:**

Our model provides a multi-scale, systems-based approach to evaluate the broader implications of therapeutic strategies.

## Introduction

A complicating aspect of the HIV-1 epidemic is that the virus spread within human populations and the course of disease within individuals are intertwined, and governed by multiple factors that cannot be easily isolated. The disease on both levels progresses through complex interactions among many components, which is typical of chronic diseases [1]. Additionally, treatment of HIV-1-infected persons affects not only their personal health, but also the degree of exposure and transmission risk to their sexual partners, and consequently the epidemic dynamics of the entire population. Therefore, a quantitative analysis of how phenomena on higher scales emerge from processes on lower scales is crucial to understanding disease dynamics. Addressing these issues requires integrated models capable of examining not only isolated factors, but also their interrelationships across relevant scales.

Traditional epidemiological modeling is based on two premises: (1) individuals within a population at any given time can be either susceptible to disease, infected, or not susceptible; and (2) the population is fully mixed. It is known that neither assumption is inadequate for modeling the HIV-1 epidemic; the first one does not account for complexity of chronic diseases with multiple stages, and the second is a poor representation of sexual contact patterns [2-26]. Here a framework combining network-type contact models with more sophisticated disease models is needed [8,10,27-32].

 We introduce a new model of HIV-1 transmission that allows for assessment of processes on multiple scales of disease, incorporating an actual sexual contact network, and a detailed model of disease progression driven by variable within-host processes, their outcomes, and medical intervention. The model is based on an MSM (Men who have Sex with Men) sexual network documented in Colorado Springs, USA [33], chosen for several reasons. First, the Colorado Springs sexual network has been well documented and the network structure is known. Second, its main core remained largely unchanged for about 5 years, hence a static network can be assumed. Third, the HIV status of approximately 70 % of the main core is known, which allows for model validation under no-treatment scenario. While simulations in this study have been performed for an MSM group, the model can be modified for general populations given that the sexual contact network is known. 

Using this model, we investigate the impact of ART initiation timing on HIV-1 spread in a population. While ART has been widely accepted as the gold standard of modern care, its timing is debated [34-39]. The current recommendation by the Department of Health and Human Services Office of AIDS Research Advisory Council [40] calls for ART initiation during the asymptomatic phase, as the number of the CD4^+^ cells of the immune system drops below 200 cells/µl. However, several recent studies provided evidence that initiating therapy earlier may offer a number of benefits such as decreased severity of symptoms during the acute infection phase; preservation of the immune system; reduction of virus diversity, and boosting the initial host response to viral replication [14,39,41-44]; reduction of seeding in latent virus reservoirs [44]; lower levels of latently infected cells [14,45,46]; reduced AIDS and death rates [47]. The downsides of early treatment initiation are the expense, long-term metabolic effects, treatment fatigue, patients not being emotionally ready to be adherent. The issue of ART timing is thus an important example of difficulties associated with decision-making in complex chronic diseases. 

Our overall aim is to evaluate the impact of medical interventions at the level of individuals, on the spread of infection across the whole population. Specifically, we investigate the impact of ART initiation timing on HIV-1 spread in an MSM population. We demonstrate that our multi-scale, systems-based approach can be successfully used to evaluate the broader implications of therapeutic strategies. 

## Methods

### Overall structure of the model

**Figure 1:**
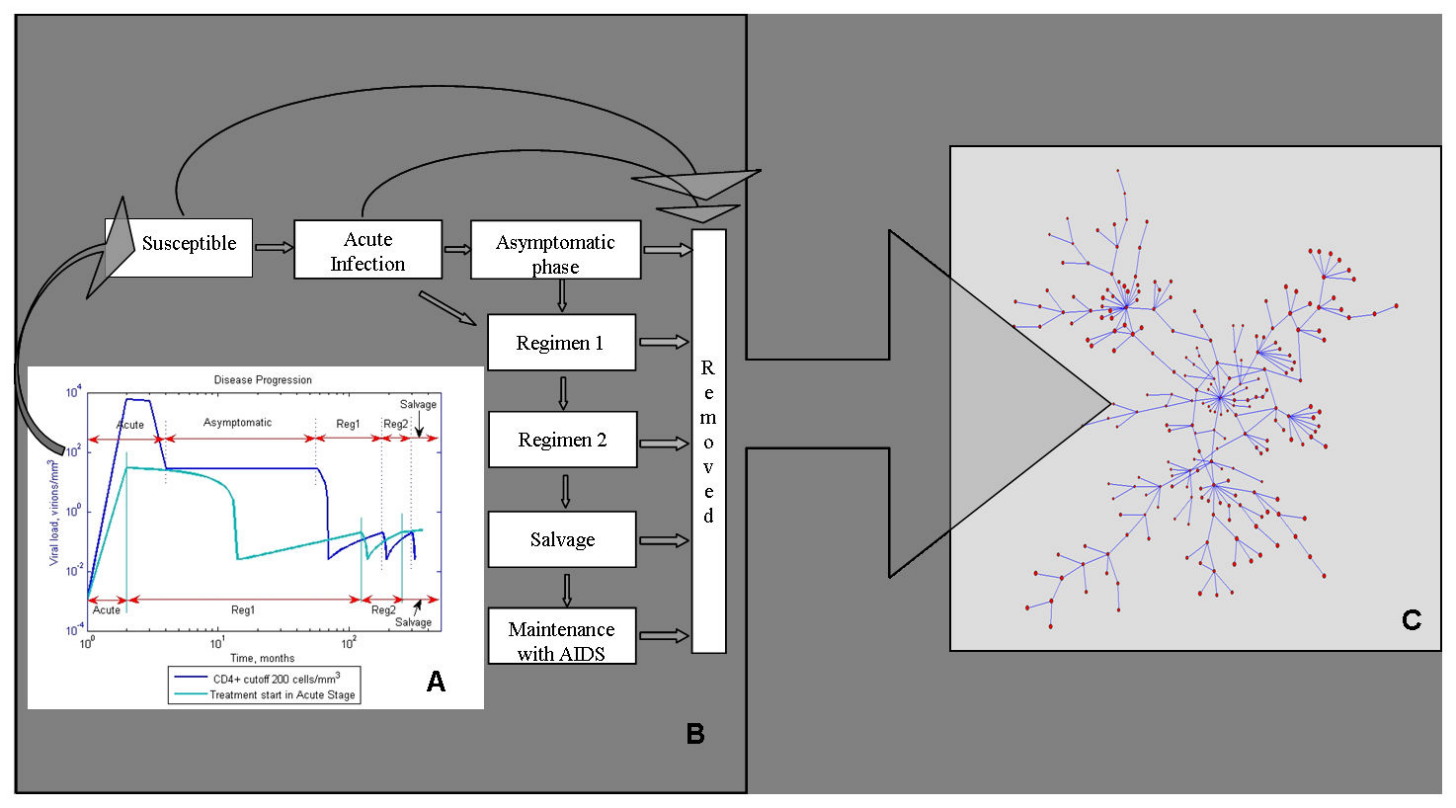
A multi-scale model of HIV-1 transmission. Within-host models (A) specific to each stage of disease (B) produce estimates of viral load and CD4+ cell count in every individual. These are key parameters that determine the course of disease, and dictate when regimens need to be changed. Graphs in (A) are a sample simulation of viral load within one patient, they demonstrate the differences in viral load in response to changing the timing of ART initiation . Viremia is tempered with ART initiated during the acute infection phase. Each individual is placed on a sexual network (C) with a distinct structure. The transmissibility is a function of the CD4+ count that determines the overall health of an individual and therefore the level of sexual activity; and of viral load which determines the probability of infection in each sexual encounter.

The model describes processes evolving on three scales: (1) within-host, (2) patient disease progression and state of health, and (3) disease transmission in a MSM population (Figure 1). The within-host scale describes dynamics and interactions between the immune system and the virus for each individual (Figure 1A). The number of CD4^+^ cells and the viral load are computed at a monthly time step, the details of computation are provided in the section “Within-host models for each health state”. If the CD4^+^ count and the viral load satisfy certain criteria, described in the section “Within-host models for each health state”, the patient transitions to a new health state. There is a total of eight health states for each individual, and the transitions between states take place at the second scale of the model (Figure 1B). Each individual is placed on a sexual contact network, which is the third scale of the model (Figure 1C). At each time step, the CD4+ count and the viral load is assessed for each individual, and his HIV-1 transmissibility is computed. Based on the transmissibility, his susceptible partners can become infected and enter the Acute Infection phase. Their disease histories then progress according to Figure 1B. 

There are several assumptions made in the model. The sexual contact network is assumed to not change over the course of simulations. As detailed below, this assumption is based on a well-documented study by [33] that indicated that this particular connected component of a larger network remained unchanged for several years during their study. Model parameter values have been obtained from several literature sources, documented in Table 1. Further, percent of individuals to receive ART was determined *a priori*. We conducted three sets of simulations, in which the probability of any individual to receive ART was set at 0, 0.3 and 1. Zero probability of receiving ART corresponds to the early period when ART was not available; probability of 1 is an extreme, ideal case of everyone receiving ART; and probability of 0.3 represents a more realistic scenario that reflects CDC’s overall epidemiologic estimate that in the USA about a third of people with HIV are taking ART regularly [48].

**Table 1:** Model Parameters.

Parameter definition	Symbol	***Value***	***Reference***
General Mortality Rate	GenMortalityRate	0.0012	[86]
Treatment Failure Rate	TreatmentFailureRate	0.0025	[54]
Infected CD4^+^ count at time zero	InitialInfCD4	0	
Viral Load at time zero	InitialViralLoad	10^-3^ viral particles mm^-3^	[57]
Uninfected CD4^+^ count at time zero	InitialUninfCD4	N (700, 100) cells mm^-3^	[87]
Death rate of free virus	c	2.4 viral particles day^-1^	[57]
Rate of CD4^+^ supply from precursors	s_T_	10 cells day^-1^ mm^-3^	[57]
Rate of CD4^+^ proliferation	p_T_	3 10^-2^ cells day^-1^	[57]
Death rate of productively infected CD4^+^	δ	2.4 10^-1^ cells day^-1^	[57]
Number of free virus produced by lysing a CD4^+^ cell	N	1200 viral particles	[57]
Death rate of uninfected CD4^+^	d_T_	2.4 10^-1^ cells day^-1^	[57]
Rate constant of CD4^+^ infection by free virus	k	2.4 10^-5^ cells mm^-3^ day^-1^	[57]
Set Point	M_p_	LogN (9.22, 1.8) viral particles mm^-3^	[53]
Probability of improvement on Regimens 1 and 2	p_EventImpr	0.47	[61]
Probability of CD4^+^ increase under viral suppression	p_EventCD4	0.87	[64]
Probability of improvement on salvage regimen	p_EventImprAfterFailure	0.38	[62]
Rate of viral load increase if therapy unsuccessful	AddRateVLIncrease	1.6 10^-3^ viral particles month^-1^ mm^-3^	[65]
Rate of CD4^+^ increase if therapy successful	RateCD4Increase	13.33 cells month^-1^ mm^-3^	[61]
Rate of viral load decrease if therapy successful		Baseline to undetectable in 2 months	[88]
Rate of viral load increase with AIDS	RateVLIncreaseAfterFailure	3.4 10^-4^ viral particles month^-1^ mm^-3^	[66]
Rate of CD4^+^ decrease with AIDS	RateCD4DecreaseAfterFailure	5 cells month^-1^ mm^-3^	[66]
Rate of CD4^+^ decline in asymptomatic phase	RateCD4DecreaseAsymptom_1, 2, 3, 4, 5	3.02 if V_sp_ ≤ 0.25	[53]
		3.73 if 0.25 < V_sp_ ≤ 1.5	
		4.6 if 1.5 < V_sp_ ≤ 5	
		5.4 if 5 < V_sp_ ≤ 15	
		6.37 if V_sp_ > 15	
Mortality rate on ART	MortalityRateOnART_1, 2, 3, 4, 5, 6	0.0016 if CD4^+^ < 25	[67]
		0.0013 if 25 ≤ CD4^+^ < 50	
		0.003 if 50 ≤ CD4^+^ < 100	
		0.0041 if 100 ≤ CD4^+^ < 200	
		0.0032 if 200 ≤ CD4^+^ < 350	
		0.0031 if CD4^+^ ≥ 350	
Number of unprotected insertive anal intercourse (UIAI) acts per month	NumberUIAI_1, 2, 3, 4, 5, 6, Acute	0.081 if CD4^+^ ≤ 50	[69]
		1.45 if 50 < CD4^+^ ≤ 100	
		0.55 if 100 < CD4^+^ ≤ 200	
		2.69 if 200 < CD4^+^ ≤ 300	
		1.11 if 300 < CD4^+^ ≤ 500	
		1.25 if CD4^+^ > 500	
		1.87 if in Acute Infection phase	
Transmission probability per act of UIAI (transmissibility)	TrPr_1, 2, 3, 4, 5, Acute	0.0008 if Viral Load ≤ 0.25	[69]
		0.0088 if 0.25 < Viral Load ≤ 1.5	
		0.0096 if 1.5 < Viral Load ≤ 5	
		0.011 if 5 < Viral Load ≤ 15	
		0.065 if Viral Load > 15	
		0.065 if in Acute Infection phase	

Below we present the model in more detail, starting with scale 2, namely the patient disease progression and state of health. This section is followed by description of scale 1, the within-host processes associated with each health state. These processes drive transitions between health states. The disease transmission model in an actual MSM population is then presented (scale 3). Finally, we discuss the uncertainty and sensitivity analysis of model outcomes.

### Disease progression and health states

States of the disease progression model correspond to clinically defined disease stages (Figure 1B). Acute infection is marked by lack of immune responses and uncontrolled viral replication with high viral loads and relatively large amounts of genital shedding of HIV-1 [49,50]. Viremia rises rapidly to a peak, then declines as the immune system starts to exert partial control [51]. In the chronic asymptomatic phase of infection the viral load reaches a quasi-steady state called the set point, which varies between persons and which determines a relatively constant rate of CD4^+^ T cell decline. If left untreated, the onset of clinical AIDS characterized by opportunistic infections and/or CD4^+^ T cell count less than 200 cells per µl of blood, takes place 4-10 years after the infection [52], but the standard of care is the institution of ART before this point.

Medical treatment significantly extends the duration of the asymptomatic phase [53]. However, under ART pressure the virus mutates and eventually becomes resistant to applied therapy. As a result, the virus load begins to steadily increase, and the treatment must be modified [54]. ART can be initiated at any time from acute infection through the asymptomatic phase in this model. Treatment affects both the viral load and CD4^+^ T cell count, therefore altering the transmission probability [50]. 

Four consecutive regimens are modeled and assigned typical rates of response with disease stabilization (reconstitution of CD4^+^ T cells and reduction of viral load to undetectable levels), and failure over time leading to the next treatment. 

The model allows for simulation of treatment scenarios. If treatment is initiated in the acute infection phase, patients move directly to Regimen 1, skipping the asymptomatic phase. Otherwise patients enter the asymptomatic phase before Regimen 1 is initiated. The model is easy to modify as more regimens become available, or as their efficacy changes.

As individuals die of various causes, they enter the Removed state. Probabilities of transitions to the Removed state correspond to mortality rates, which vary depending on the state of health [55,56].

### Within-host models for each health state

For each individual we trace two key parameters: the viral load and the CD4+ cell count. The coupled dynamics of virus and CD4+ populations drive the transitions between disease stages (Figure 1B). Figure 1A demonstrates a simulation sample of viral load evolution during different stages of disease progression, under two treatment initiation scenarios. The viral load is typically lower in the case of early ART initiation. Below we describe the within-host models of viral and CD4+ population dynamics used in generating Figure 1A.

#### Acute infection

Healthy individuals are at risk of acquiring HIV-1 if their sexual partners are infected. Once infected, an individual enters the acute infection phase. The acute infection phase is modeled after Perelson and Nelson [57]. Three cell populations are considered: uninfected CD4^+^ cells *T*, productively infected CD4^+^ cells T* and free virus *V*: 

$$\begin{array}{c} \frac{dT}{dt}=s+pT(1-\frac{T}{T_{max}})-d_{T}T-kVT\\ \frac{dT^{*}}{dt}=kVT-\delta T^{*}\\ \frac{dV}{dt}=N\delta T^{*}-cV \end{array}$$ (1)

The definitions and values of parameters are in Table 1. Equations (1) are solved numerically in MatLab over 10 weeks after the infection event, which is a typical duration of the acute infection phase. At 10 weeks the simulation for the acute infection stage is terminated and the patient enters the asymptomatic stage.

#### Asymptomatic phase

The asymptomatic phase is treated as the period where the CD4^+^ count decays linearly in untreated patients, with slope *m* determined by the log-normally distributed set point logN (9.22, 1.8) [53]. The viral load remains constant at the set point level, as there is a quasi-steady state during the chronic asymptomatic phase of infection [58,59,60]. Transition to Regimen 1 takes place when the CD4^+^ count falls below a predetermined threshold.

#### Treatment regimens

Clinicians treating patients have choices between many treatment regimens.  Generally, all commonly utilized treatment regimens include a combination of three drugs: two nucleoside reverse transcriptase inhibitors (NRTIs) and a non-nucleoside reverse transcriptase inhibitor (NNRTI), or two NRTIs and a protease inhibitor (PI) [40].  These are considered "first line therapies" with equal efficacy.  Given the number of medications available, it is usually possible to have two first line therapies plus a maintenance regimen that do not overlap in drug resistance patterns, and patients can use these therapies sequentially without loss of efficacy if they have failed their first treatment regimen due to factors such as poor adherence and development of drug resistance [61].  Following regimens are considered "second line" and necessarily include re-use of dual NRTIs for which some resistance mutations have occurred during the first and second regimens, and therefore have lesser efficacy.  These are often described as "salvage" or "rescue" regimens [62].

Regimens 1 and 2 of our model correspond to the first line therapies. For either regimen, each patient is assumed to have 0.47 probability of suppressing the virus, i.e. improving on a given regimen [61,63], about 87% of these patients experience restoration of CD4^+^ counts [64]. The monthly rate of CD4^+^ increase is about 13.33 cells/mm^3^ [61]. 

In patients not responding well to ART, the inability of drugs to suppress the viral load leads to its steady increase at a monthly rate of about 0.0016 virions/mm^3^ [65]. Once the viral load exceeds 0.2 viral particles/mm^3^, the regimen has failed, and the next regimen is initiated. 

The salvage regimen is less efficacious than the first two: viral load suppression probability is 0.38 [62]. Changes in the CD4^+^ count and viral load are calculated as before. Failure of the salvage regimen leads to increased incidence of opportunistic infections and placement of patients on a “maintenance” regimen [66,67,68]. While on maintenance therapy, the viral load increases at a steady rate of 3.4 10^-4^ viral particles/mm^3^/month, while the CD4+ count decreases at 5 cells/mm^3^/month [66]. Mortality rates of patients on ART, i.e. probabilities of transitioning to state Removed, are documented in [55] (Table 1). 

### Scenarios of interventions tested

 HIV-1 spread was simulated over a period of 3 years, with a monthly time step, i.e. all parameters and transitions from one health state to another were updated once a month. This time frame was chosen to satisfy the static network assumption [33], while allowing sufficient time to study the temporal spread of disease. 

Three scenarios were simulated, testing the effects of initiating ART at different stages of disease (Figure 1B). The first scenario corresponds to starting ART when CD4^+^ T cell count drops below 200 cells/mm^3^; this is the only treatment strategy that has been shown to definitively improve survival in clinical studies to date. The second scenario corresponds to current treatment guidelines of starting ART when CD4^+^ T cells drop below 350 cells/mm^3^ and/or the viral load exceeds 50 viral particles/mm^3^ during the chronic asymptomatic phase, which is based on consensus expert opinion [40]. The third scenario is the debated strategy of initiating ART during acute infection [35]. The impact of these medical strategies on HIV-1 spread is analyzed.

Percent of individuals to receive ART was determined *a priori*. We conducted three sets of simulations, in which the probability of any individual in the acute infection stage to receive ART was set at 0, 0.3 and 1. In case a patient was chosen to receive ART, he would go through all regimens from regimen 1 to maintenance (Figure 1B), with ART initiated according to the scenario being simulated. 

### Transmission model

#### Transmission characteristics

At the level of individuals, we utilize two key clinical parameters to determine if transmission takes place: CD4^+^ T cell count is the key predictor of health status [40] and therefore of likelihood to engage in sexual activity [69]; and viral load is an indicator of HIV-1 shedding and risk of transmission during sexual contact [14,69]. In each contact, transmission occurs with probability equal to the product of sexual intercourse frequency and risk of transmission, which are tabulated as functions of CD4^+^ count and viral load, respectively (Table 1). Intercourse frequency is expressed as the number of unprotected insertive anal intercourse (UIAI) acts per month, which leads to the highest exposure to HIV-1 [69].

#### Network of sexual contacts

The model was validated with the sexual network originally described by Potterat et al, based on the analysis of community-wide contact tracing records [33]. The dataset contained 1933 partners, out of which 250 belonged to the largest connected component during the first five years of the study. We use this component for our simulations, as it remained virtually unchanged during that time and we could safely assume that the network was static in the sense that the edges representing sexual contacts remained the same. From the 250 subjects, we retained 231 homosexual and bisexual males and excluded 19 females and heterosexual males. These 19 individuals were on the periphery of the network, and their removal did not break the component. The network structure and information was obtained from Figure 2A of [33], and re-drawn in Figure 1C with Pajek software [70]. The distribution of the number of sexual partners in this community was uneven: most individuals had less than 5 sexual partners, while two had 20 partners each. At the time of data collection, about 41% were positively diagnosed for HIV, 34% were negative and the HIV status of 25% was not known[33,70].

**Figure 2:**
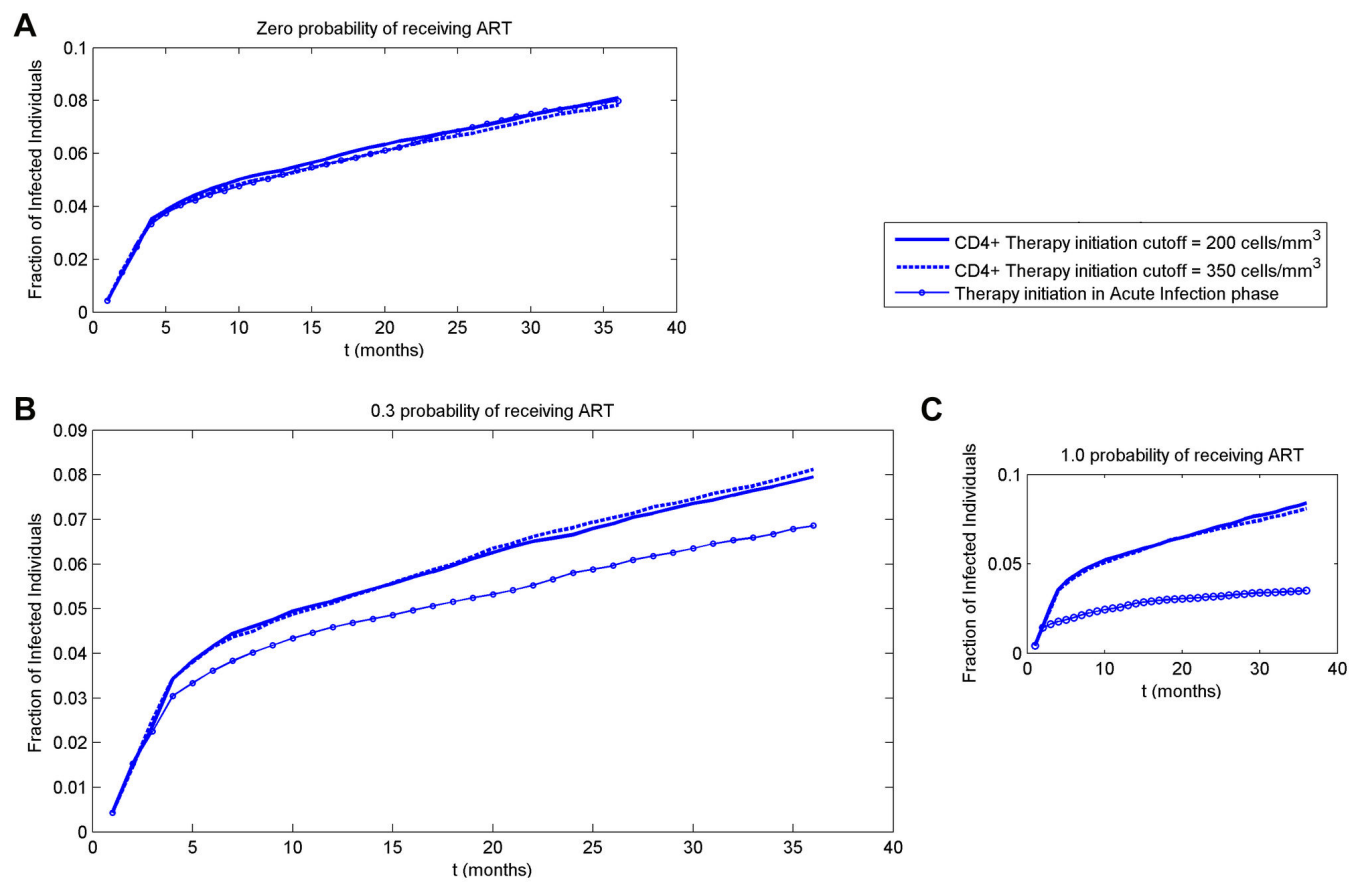
Temporal propagation of disease within 3 years for three scenarios of ART initiation timing. In all simulations a patient with 20 sexual partners is infected at time zero. Three scenarios of ART initiation are simulated: at the CD4^+^ cutoff below 200 cells/mm^3^, 350 cells/mm^3^ and in acute infection phase. (A) No one receives ART; (B) about 30% of infected individuals receive ART; (C) all infected individuals receive ART.

#### Uncertainty and sensitivity analysis

Special numerical methods are needed to analyze the behavior of complex mathematical models such as ours, as well as the effect of model parameters on its output. Uncertainty analysis assesses the variability of model outcomes due to uncertainty in estimation of input parameters, while sensitivity analysis extends the uncertainty analysis by evaluating the importance of input parameters [71]. Both are powerful tools designed to investigate the behavior of complex models with uncertain input parameters [72]. 

We used the Latin Hypercube Sampling (LHS) scheme, one of the most computationally efficient methods for uncertainty analysis [72,73,74]. In this method, input parameters are treated as random variables, each with its own probability distribution function. Parameter values are chosen randomly from their respective stratified distribution functions; input vectors are thus created containing parameter values for model simulations. The model is then run several times, each time with a new set of input parameter values, and its output is recorded. This completes the uncertainty analysis; next the sensitivity analysis is performed, here with factor prioritization by reduction of variance used to rank the importance of parameters to model outcomes. This method is applied when the relationship between the outcome and input parameters is highly nonlinear, which is the case here [75]. Sensitivity indices produced for each input parameter are interpreted as the proportion of total output variance attributable to the given parameter [72]. We implemented the analysis with MatLab-based SaSAT software [75]. 

## Results

### Epidemiology

The temporal propagation of disease for varying probability of receiving ART, p_ART_ = 0, 0.3, 1 is shown in Figure 2. The case of p_ART_ = 0 refers to years before introduction of ART; p_ART_ = 0.3 represents an assumption that currently about 30% of infected individuals receive ART; p_ART_ = 1 represents an ideal situation where ART is received by all infected individuals. 

In each case, all three scenarios of ART initiation timing are tested, with 100 simulations performed for each scenario. At time zero, one of the two individuals with the highest number of sexual contacts (twenty) is infected, while the rest of the population is healthy and therefore susceptible to disease. When no ART is available to anyone (Figure 2A), all three scenarios converge into the same curve, as expected. At the end of the 3-year simulation, about 8% of the population is infected. With p_ART_=0.3 and ART initiation in the acute infection phase, at 3 years less than 7% of population are infected (Figure 2B); with p_ART_=1, about 3% are infected (Figure 2C). The reduction of 0.01 in fraction infected at the end of the simulation (Figure 2B, p_ART=0.3) translates into a maximum of about 7.5 patient-years over 3 years. This number would then translate into a respective reduction in ART medication for the whole population. Collectively, these results indicate that significant reduction in the fraction of infected individuals could be achieved by initiating ART as early as possible, and by administering it to as many individuals as possible.

The results also indicate that initiating ART at CD4^+^ T cell counts of 200 versus 350 cells/mm^3^ demonstrates minimal difference in the spread of HIV-1. For p_ART_=1 (Figure 2C), the difference is about 8.4 versus 8.1% at 3 years, while ART initiation during acute infection markedly decreases the fraction of infected persons (to about 3.5% at 3 years). A similar picture arises in p_ART_=0.3 (Figure 2B). Therefore, modifying ART initiation threshold during chronic infection (first two scenarios) had a small effect at the population level. 

**Figure 3:**
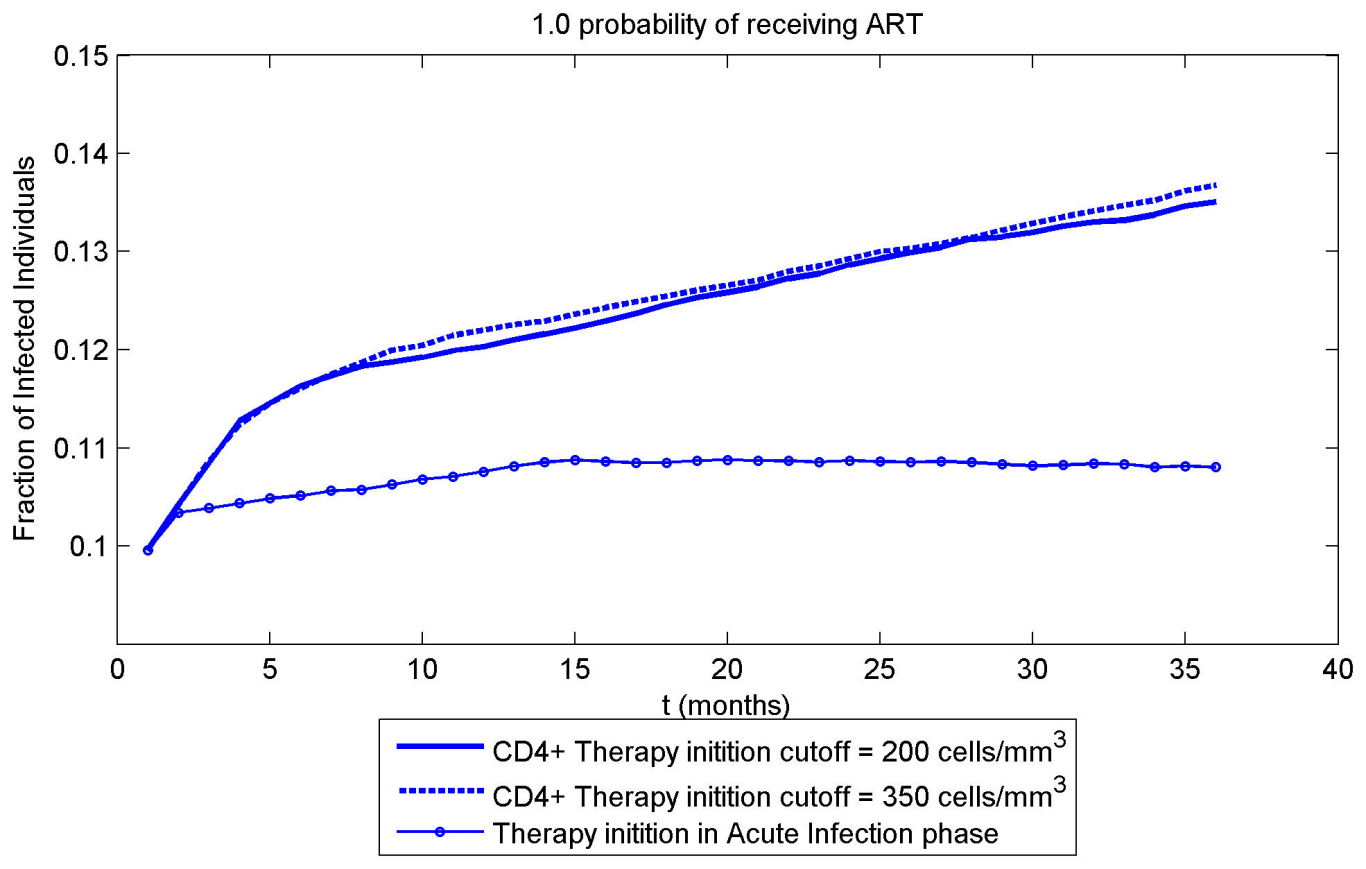
A cluster containing 10% of the population is infected at time zero, all infected individuals receive ART. ART initiation scenarios are as in Figure 2. The simulation is performed to test the response of the model to an increase of the number of patients infected at time zero.

The number of patients infected at time zero is an important factor in the dynamics of disease spread. We ran a simulation with a larger initial number (about 10% of entire population) of infected individuals (Figure 3). In this case a much higher fraction of individuals are infected at 3 years in all scenarios, as expected. Similarly to the case of only one highly connected individual infected at time zero (Figure 2C), ART initiation during acute infection blunts the spread of the virus compared to later ART initiation (10.8% for early ART versus 13.5% and 13.7% for ART starting at CD4^+^ T cell counts of 200 and 350). Notably, for both initial conditions, there is an early phase of rapid HIV-1 spread that is particularly suppressed by early ART, likely due to aggressive early spread of disease by contagious persons with acute infection (high viral loads). Thus, ART initiation during the acute infection phase significantly alters the course of the epidemic, and results in much lower fractions of infection in the long term. 

**Figure 4:**
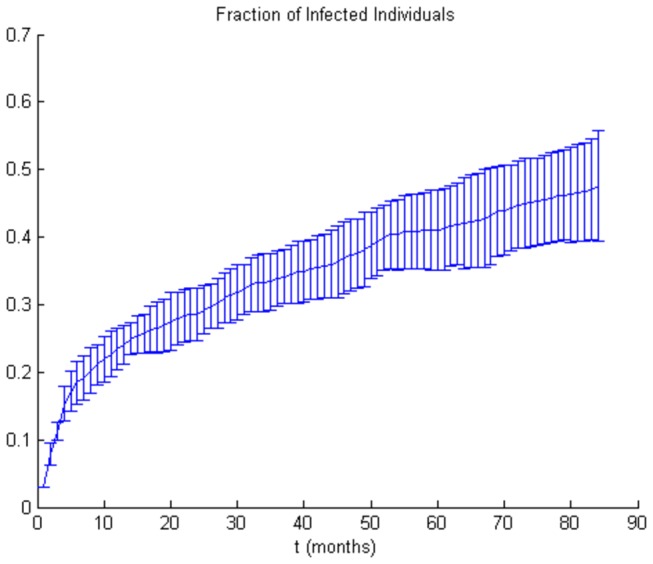
The two individuals with the highest number of sexual partners (20) are infected at time zero. No one receives ART. The simulation is performed to test the response of the model to the impact of highly connected individuals.

To test the model predictions we simulated a situation with no ART available to anyone, and with both highly connected individuals infected at time zero (Figure 4). This situation is likely to have arisen in Colorado Springs in the mid 80s, when no ART was available. Simulations indicate that at 5 years the fraction of infected individuals averaged to about 40%, which is in excellent agreement with data from Colorado Springs where 41% are HIV positive [33]. 

Our results also agree with the conclusions of a recent study by Granich and co-workers [36] who looked at possible outcomes of universal HIV testing with immediate initiation of ART. Their model was based on general population data from South Africa. Similarly to our results (Figure 4), the authors predicted a 6% increase in new infection cases in the course of a year in a scenario without ART. They also modeled a scenario in which individuals would be tested on a yearly basis and, if tested positive, would immediately be offered ART. In this scenario not all newly diagnosed individuals would still be in the acute infection phase and many would have progressed into the asymptomatic phase, however the results generally agree with our conclusions. Our model predicts a 56% drop at 3 years in the fraction of infected individuals who are offered ART in the acute infection phase (Figure 2C) as opposed to those who are not offered ART (Figure 2A). Granich and co-workers calculated a 45% difference in HIV prevalence between no ART and immediate ART initiation upon positive HIV test scenario. This re-affirms that starting ART in the acute infection phase is likely to be highly efficient in curbing the spread of disease.

### Uncertainty and sensitivity analysis

Uncertainty and sensitivity analysis were conducted for all three scenarios of ART timing initiation; for each scenario 200 simulations were performed. The model output is the fraction of infected individuals; parameter values are sampled from respective probability distributions (Table 2). The results are shown in Table 3. In both scenarios of late ART initiation, the parameter responsible for most of disease spread variability is Mp, the viral set point; it accounts for 94% and 86.6% of model output variability in cases of ART initiation below CD4^+^ count of 200 cells/m^3^ and 350 cells/m^3^ respectively. The contribution of the rest of model parameters is negligible (less than 2% each in either scenario). 

**Table 2:** Parameter distributions for uncertainty and sensitivity analysis.

**ParameterName**	**Distribution^1^**	**Argument 1^2^**	**Argument 2**
GeneralMortalityRate	U	5.75 ⋅ 10^-5^	1.73 ⋅ 10^-4^
TreatmentFailureRate	U	0	5 ⋅ 10^-3^
CD4AsymptomPhaseCutoff	D	2 ⋅ 10^2^	
InitialUninfCD4	N	7 ⋅ 10^2^	10^2^
InitialInfCD4	D	0.00	
InitialViralLoad	U	5 ⋅ 10^-1^	5 ⋅ 10^6^
TrPr_Acute	U	4.4 ⋅ 10^-2^	8.6 ⋅ 10^-2^
TrPr_1	U	5.4 ⋅ 10^-4^	10^-3^
TrPr_2	U	5.9 ⋅ 10^-3^	1.2 ⋅ 10^-2^
TrPr_3	U	6.4 ⋅ 10^-3^	1.3 ⋅ 10^-2^
TrPr_4	U	7.4 ⋅ 10^-3^	1.5 ⋅ 10^-2^
TrPr_5	U	1.2 ⋅ 10^-2^	2.4 ⋅ 10^-2^
NumberUIAI_1	G	7.1 ⋅ 10^-1^	2.65
NumberUIAI_2	G	7.1 ⋅ 10^-1^	1.15
NumberUIAI_3	G	7.1 ⋅ 10^-1^	2.05
NumberUIAI_4	G	7.1 ⋅ 10^-1^	7.8 ⋅ 10^-1^
NumberUIAI_5	G	7.1 ⋅ 10^-1^	3.80
NumberUIAI_6	G	7.1 ⋅ 10^-1^	1.57
NumberUIAI_7	G	7.1 ⋅ 10^-1^	1.77
MortalityRateOnTreatment_1	U	8 ⋅ 10^-4^	2.4 ⋅ 10^-3^
MortalityRateOnTreatment_2	U	6.5 ⋅ 10^-4^	1.9 ⋅ 10^-3^
MortalityRateOnTreatment_3	U	1.5 ⋅ 10^-3^	4.5 ⋅ 10^-3^
MortalityRateOnTreatment_4	U	2.5 ⋅ 10^-3^	6.1 ⋅ 10^-3^
MortalityRateOnTreatment_5	U	1.6 ⋅ 10^-3^	4.8 ⋅ 10^-3^
MortalityRateOnTreatment_6	U	1.5 ⋅ 10^-3^	4.6 ⋅ 10^-3^
p_eventImpr	U	10^-1^	6 ⋅ 10^-1^
p_eventCD4	U	5 ⋅ 10^-1^	1.00
AddRateVLDecrease	U	10^-3^	3 ⋅ 10^-3^
RateCD4Increase	U	6.67	20.0
AddRateVLIncrease	U	8 ⋅ 10^-4^	2.4 ⋅ 10^-3^
c	U	1.20	3.60
sT	U	1.5 ⋅ 10^2^	4.5 ⋅ 10^2^
pT	U	4.5 ⋅ 10-^1^	1.35
Tmax	U	7.5 ⋅ 10^2^	2.25 ⋅ 10^2^
dT	U	3 ⋅ 10^-1^	9 ⋅ 10^-1^
k	U	3.6 ⋅ 10^-4^	1.1 ⋅ 10^-3^
delta	U	3.60	1.08 ⋅ 10^1^
N	U	6 ⋅ 10^2^	1.8 ⋅ 10^3^
Mp	LN	9.22	1.80
p_eventImprAfterFailure	U	0.00	5 ⋅ 10^-1^
RateVLIncreaseAfterFailure	U	1.7 ⋅ 10^-4^	5.1 ⋅ 10^-4^
RateCD4DecreaseAfterFailure	U	2.50	7.50
RateCD4DecreaseAsymptom_1	U	1.51	4.53
RateCD4DecreaseAsymptom_2	U	1.87	5.60
RateCD4DecreaseAsymptom_3	U	2.30	6.90
RateCD4DecreaseAsymptom_4	U	2.70	8.10
RateCD4DecreaseAsymptom_5	U	3.19	9.56
p_ART	N	4 ⋅ 10^1^	10

1 U(Uniform), N(Normal), G(Gamma), D(Deterministic)

2 For Uniform distribution, Argument1 is the minimum, Argument 2 is the maximum of the range

For Normal and LogNormal distributions, Argument1 is the mean, Argument 2 is the standard deviation

For Gamma distribution, Argument1 is the shape parameter, Argument 2 is the scale parameter

**Table 3:** Sensitivity indices (above 0.05 in bold) representing proportion of total variance attributable to the given parameter.

**Parameter**	**Scenario 1: Treatment start at CD4≤200**	**Scenario 2: Treatment start at CD4≤350**	**Scenario 3: Treatment start in Acute Infection**
Mp	**0.94**	**0.86**	**0.19**
p_eventCD4	0.000066	0.0002	**0.077**
NumberUIAI_5	0.002	0.006	**0.069**
MortalityRateOnTreatment_3	0.005	0.002	**0.065**
NumberUIAI_6	0.002	0.01	**0.064**

In the case of ART initiation in the acute infection phase, the viral set point remains the most influential parameter, albeit with a much lower sensitivity index at 19.1%. It appears that early ART initiation blunts the large effect of the viral set point. Its contribution to the output variability is balanced by four other parameters: p_eventCD4, the probability of CD4^+^ increase under viral suppression (7.7%); MortalityRateOnTreatment_3, the mortality rate while on ART, with CD4^+^ count between 50 and 100 cells/mm^3^ (6.5%); and the number of UIAI acts per month with the CD4^+^ count above 500 cells/mm^3^ (6.9%) and during the acute infection phase (6.4%). The contribution of each of the remaining parameters to the spread of disease is below 5%.

The viral set point is a major indicator of the course of disease following the end of the acute infection phase, and is highly correlated with the CD4^+^ count decay in the asymptomatic phase and with viral activity [53]. So, it is not surprising that in cases of late ART initiation when the disease develops according to its natural pace for an extended duration of time, the viral set point plays a major role. The CD4^+^ count is a general indicator of an individual’s health state and of sexual activity [69] (Table 1), hence the significance of p_eventCD4. The importance of UIAI acts per month, particularly during acute infection, is in accord with evidence that as high as 48% of transmission cases take place during the acute infection phase [76], and with our results demonstrating the difference that early ART initiation makes in disease propagation (Figure 2).

## Discussion

Multi-scale modeling provides a possibility for studying relationships between processes on different scales within the same framework. The integration of models at microbiologic, individual host, and population scales offers a medium to examine how the effects of individual interventions scale across the entire framework. Our model takes this approach to capture how the dynamics of disease progression in individuals affect HIV-1 spread within an MSM population. This allows for simulation of medical interventions, such as varying treatment strategies, drug efficacies, and prevention strategies. For example, this modeling framework would be suited for evaluating the impact of interventions such as the effect of ART initiation upon detection of HIV during once-yearly testing and treatment, behavioral changes, implementation of a vaccine that reduces viral load without preventing infection, and modification of community-wide treatment recommendations.

We have tested the model in simulations of altering the ART initiation timing, a topic of current controversy [34,35,39]. The problem of treatment initiation timing is an excellent example of intricacies of decision-making in complex diseases that evolve on multiple scales. On the one hand, deferring ART would delay the development of drug resistance and preserve therapeutic options, reduce the accumulated effects of drug toxicity, and conserve potentially limited resources. On the other hand, the longer period off treatment could render infected persons more contagious and allow greater spread of virus. Our simulations, utilizing several biologically observed parameters, have addressed this latter factor by estimating how different interventions on the microbiologic scale affect population-wide spread of HIV-1. These simulations suggest that altering the timing of ART initiation during chronic infection has a minimal impact on community transmission (suggesting that patients can be saved the misery of the heavy ART medication, and society the costs of additional ART by not treating at 350 CD4^+^ cells/mm^3^), but that routinely starting ART during acute infection could have a major role in reducing spread.

These results are encouraging support for our model, in light of several clinical observations regarding the immunopathogenesis of HIV-1 infection. Potent ART regimens developed since the mid-1990s are successful at suppressing viral replication within infected persons, thereby halting or reversing disease progression and extending the asymptomatic phase of infection in association with reducing viral replication as measured by viral load [14,15]. Viral replication is also a key factor in host-host transmission of HIV-1; and viral load is a strong predictor of the risk of transmission per exposure. Acute infection is characterized by extremely high viral loads and virus shedding in genital secretions [49,50], and this probably explains the observation that acutely-infected individuals contribute disproportionately to HIV-1 spread, with an 8 to 20 fold increase in odds of transmission per coital act [14]. It is estimated that as high as 48% of all cases of HIV-1 transmission occur when the transmitting host is within the first five months of being infected [76]. Thus, our model yields results that are compatible with the biological principles of HIV-1 infection, suggesting that suppression of viral replication with ART in the early stage of infection mediates a disproportionate effect in preventing transmission compared to starting ART during chronic infection. This is consistent with a hypothesized benefit of early treatment in attenuating the spread of HIV-1 [14,26,49,50,76,77,78]. Several recent studies also concluded that universal and if possible early administration of ART is a powerful way to prevent the spread of the disease [36,37,79]

A major obstacle to early implementation of ART is the timely diagnosis of early HIV-1 infection, primarily due to absence of specific and recognizable symptoms. To aggravate the problem, routine HIV-1 antibody tests remain negative for several weeks past initial infection. More sensitive, virus-specific tests such as HIV p24 antigen ELISA and HIV nucleic acid amplification assays are available but they are expensive and prone to high (1%) false-positive rates. 

Strategies however can be and are being developed to meet these challenges. The blood-banking industry routinely combines antibody and HIV RNA tests, such as initial screening of specimens using antibody testing followed by blood pooling prior to HIV RNA testing. These strategies are being extended to clinical testing in Swiss, US and Indian studies. 

Interestingly, acute HIV-1 infection is not rare in clinical-testing groups [80,81,82], especially in hyperendemic regions such as India and Malawi [83,84]. Hence, a combination of accurate testing strategies and screening/tracing programs such as for instance “network notification” whereby partners of HIV-positive individuals are notified of their need for HIV testing [85] could potentially dramatically increase the level of acute infection detection. 

A potential development of the model would be the use of dynamic network models from following MSM or mixed communities over time. Other macro-scale issues that could be incorporated include the cost of ART and limited distribution of drugs in resource-poor locales. At the individual scale, adherence to therapy and host-specific microbiologic factors could be included. At the microbiologic scale, designating viral loads as a single determining factor with stochastic variation in set-point is an oversimplification of complex processes. Further defining lower scales such as HIV-1 evolution and mutation, host immunity, metabolism and genetic background, may be important to provide more accurate predictions of ART and vaccine efficacy, and drug toxicity.

Ultimately, the goal is for medical decision-making to significantly benefit from utilization of multi-scale models. Such models are particularly needed for chronic infectious diseases such as HIV-1 infection, where individual medical interventions are likely to have effects on the pattern of the epidemic, and where public policy decisions are likely to affect individuals. Thus a systems-based approach is crucial to gauge and predict benefits and risks across all scales. Our proposed model has potential applications where such multi-scale considerations are important, as we have demonstrated by examining an ongoing unresolved clinical question regarding timing of ART initiation.
